# A Novel Application of Deep Learning (Convolutional Neural Network) for Traumatic Spinal Cord Injury Classification Using Automatically Learned Features of EMG Signal

**DOI:** 10.3390/s22218455

**Published:** 2022-11-03

**Authors:** Farah Masood, Milan Sharma, Davleen Mand, Shanker Nesathurai, Heather A. Simmons, Kevin Brunner, Dane R. Schalk, John B. Sledge, Hussein A. Abdullah

**Affiliations:** 1 School of Engineering, University of Guelph, Guelph, ON N1G 2W1, Canada; 2 The Department of Biomedical Engineering, Al-Khwarizmi College of Engineering, Baghdad University, Baghdad 10071, Iraq; 3 The Wisconsin National Primate Research Center, University of Wisconsin-Madison, Madison, WI 53715, USA; 4 The Division of Physical Medicine and Rehabilitation, Department of Medicine, McMaster University, Hamilton, ON L8S 4L8, Canada; 5 The Department of Physical Medicine and Rehabilitation, Hamilton Health Sciences, St Joseph’s Hamilton Healthcare, Hamilton, ON L8N 4A6, Canada; 6 The Lafayette Bone and Joint Clinic, Lafayette, LA 70508, USA

**Keywords:** deep learning, machine learning, convolutional neural network, *k*-nearest neighbors, electromyography, spinal cord injury, non-human primate

## Abstract

In this study, a traumatic spinal cord injury (TSCI) classification system is proposed using a convolutional neural network (CNN) technique with automatically learned features from electromyography (EMG) signals for a non-human primate (NHP) model. A comparison between the proposed classification system and a classical classification method (*k*-nearest neighbors, *k*NN) is also presented. Developing such an NHP model with a suitable assessment tool (i.e., classifier) is a crucial step in detecting the effect of TSCI using EMG, which is expected to be essential in the evaluation of the efficacy of new TSCI treatments. Intramuscular EMG data were collected from an agonist/antagonist tail muscle pair for the pre- and post-spinal cord lesion from five *Macaca fasicularis* monkeys. The proposed classifier is based on a CNN using filtered segmented EMG signals from the pre- and post-lesion periods as inputs, while the *k*NN is designed using four hand-crafted EMG features. The results suggest that the CNN provides a promising classification technique for TSCI, compared to conventional machine learning classification. The *k*NN with hand-crafted EMG features classified the pre- and post-lesion EMG data with an F-measure of 89.7% and 92.7% for the left- and right-side muscles, respectively, while the CNN with the EMG segments classified the data with an F-measure of 89.8% and 96.9% for the left- and right-side muscles, respectively. Finally, the proposed deep learning classification model (CNN), with its learning ability of high-level features using EMG segments as inputs, shows high potential and promising results for use as a TSCI classification system. Future studies can confirm this finding by considering more subjects.

## 1. Introduction

Traumatic spinal cord injuries (TSCI) comprise a serious public health burden. Worldwide, it has been estimated that there are approximately 930,000 new cases a year, and approximately 27 million people are currently living with TSCI [1]. Common causes include motor vehicle crashes, sports injuries, falls, and interpersonal violence [2]. Patients may experience a plethora of clinical deficits, which can be attributed to the injury of specific fiber tracts [3]. Typical symptoms include limb weakness and sensory abnormalities, in addition to bowel and bladder dysfunction [4]. TSCI has a devastating effect on the physical, psychosocial, and emotional aspects of the patient and the caregivers [2]. Although rehabilitation treatments have positively impacted patient independence, there exist limited pharmacological approaches to mediate improvements in volitional limb control [4]. In this context, the development of a valid non-human primate (NHP) model of TSCI with an efficient assessment tool may assist in the identification and evaluation of candidate therapeutic agents for this condition [4].

Electromyography (EMG) signals are bioelectrical signals generated from muscle fibers during muscle contractions [5]. Skeletal muscle is composed of densely packed muscle fibers innervated **by** motor neurons [6]. The motor fibers subtended by one motor neuron are considered a motor unit (MU). Each muscle consists of multiple MUs, which act in a graded and coordinated manner to generate a functional muscle contraction. To increase or maintain the generated force of a muscle, the MUs fire repeatedly and generate a motor unit potential train (MUPT). A detected EMG signal is composed of the superposition of the generated MUPTs of all fired MUs and any accompanying interference signal, such as background noise [7,8,9].

EMG signals were initially analyzed based on a number of spikes in the aggregate signal [4], followed by more sophisticated approaches, such as those based on wavelet analysis [10] and machine learning [11]. Consequently, in the present work, we propose a new TSCI classification system using raw EMG segments and deep learning (DL) classification through the use of a convolutional neural network (CNN). Moreover, a comparison between the proposed classification system and a classical machine learning (ML) classification technique—*k*-nearest neighbors (*k*NN)—is presented.

Despite the valuable information included in the recorded EMG signal, its complex and non-stationary nature makes the process of extracting relevant features a challenging task, particularly for a dynamic free muscle activity with different levels of contractions. Recent advances in the AI field—particularly DL techniques—may help to overcome this issue and promote new classification approaches. DL techniques are unique, in terms of their learning ability for high-level features, which eliminates the need for the challenging feature extraction process. Another key factor that makes DL more popular is the ability to be used even with complex, wide, and unstructured data [12,13]. Although growing attention has been given to the DL techniques, including CNNs, autoencoders (AEs), and recurrent neural networks (RNNs) in the EMG motor control area, to the best of our knowledge, the EMG-based classification system for neuromuscular disease is still a new application, which has not been investigated previously. Therefore, it seems reasonable to start investigating this type of learning style and compare it to conventional machine learning techniques that have been studied extensively in the literature. CNN has been used as a successful tool in various EMG motor control studies, achieving competitive results compared to other machine learning classification techniques. Table 1 summarizes the most recent applications of deep learning in the EMG classification field.

The overall objective of this research program is to design a valid NHP model of TSCI with two goals: (1) measuring the initial impairment associated with an experimentally created spinal cord lesion, and (2) measuring natural recovery without treatment and recovery with treatment. This impairment was measured with respect to electromyographic signals obtained from muscles affected by the experimental spinal cord injury. Stated in another way, the EMG can be considered a neurophysiological biomarker. This paper presents the results and analysis investigating the first goal using EMG signals with two different classification techniques (*k*NN and CNN).

## 2. Materials and Methods

### 2.1. EMG Acquisition Setup and Protocol

The experimental methods were as described in detail elsewhere [3,4,10,11,24]. Briefly, the subjects were adult *Macaca fasicularis* monkeys. All procedures were completed with a sterile technique and under anesthesia. A transmitter was inserted, which recorded the EMG signal from the tail’s left and right flexor cauda longus and brevis muscles. An experimental spinal cord lesion was created using an epidural catheter. The applied protocol in this experiment was approved by the Institutional Animal Care and Use Committee (IACUC) at Harvard University and the University of Wisconsin at Madison. This study represents a novel analysis of the data utilizing a deep learning approach.

### 2.2. EMG Data Analysis

#### 2.2.1. EMG Data Pre-Processing

In this section, the recorded raw EMG signals were filtered using a bandpass filter (fourth-order Butterworth filter with lower and upper cut-off frequencies of 10 and 450 Hz, respectively). Another filtering stage was implemented on the data using a notch filter at 60 Hz, in order to eliminate the power line noise. Additionally, the input signal was processed forward and backward, in order to resolve phase shift problems. The EMG signal conditioning phase was implemented using MATLAB software (MathWorks, Natick, MA, USA).

The filtered EMG signal for each individual day of the experiment was segmented into a series of disjoint windows of size 1000 ms. A different number of EMG segments were then tested as inputs to the classification system. After multiple trials, 53,350 pre-lesion EMG segments and 135,300 post-lesion EMG segments, combined for all the subjects, were chosen to be used as classifier inputs. This number was selected as a compromise between the improvement in the classification accuracy and the time consumed. According to the nature of the experiment, the collected data were imbalanced, as they were collected for more days during the post-lesion period (90 days), compared to the pre-lesion period (30 days). An imbalanced data set can lead to inaccurate classification results [25]. To address this problem, the data set was balanced by applying the random over-sampling technique in the pre-lesion class. The samples of the balanced data set were normalized between 0 and 1 using min–max normalization, followed by splitting into training and test sets in an 80:20 split.

#### 2.2.2. Classification Techniques

Two different styles of classification techniques were employed for comparison. These techniques are as follows:

##### kNN Classification

*k*NN is a supervised, non-parametric machine learning classification approach. It is a simple and practical algorithm which can be employed for classification and regression tasks [26]. *k*NN has been used widely in the EMG research area, and its efficiency has been proven in many EMG classification applications [27,28,29,30].

Four of the standard EMG amplitude features were extracted from the prepared EMG segments, including the area, the root mean square (RMS), the turn number, and the zero-crossing number. All features were then standardized and normalized. The created feature vector was then utilized as an input to the *k*NN classifier. Different *k* values were tested, and the best accuracy was obtained when using *k* = 9.

##### CNN Classification

I. The Main Layers of CNN

A CNN consists of multiple structural layers of various types, where the three main types of layers are as follows [31,32]:

*Convolutional layer*: This is an essential component of any CNN. It includes linear and non-linear operations (convolutional and activation functions). The convolutional layer comprises a specific number of filters (kernels) that are applied to the input data. These filters work as detectors to extract different features of the input data. Each filter convolves with input data elements (e.g., image: pixels, signal: data points) by shifting it horizontally and vertically a certain number of steps (known as the stride). All outputs from the convolution step are then combined into one volume, which is known as a feature map. Next, a non-linear function (usually a rectified linear unit, ReLU) is applied to the created feature maps. The ReLU function has been shown to be useful in solving the vanishing gradient problem during the back-propagation process [33], which helps in reducing the training time. Then, to reduce the size of the extracted feature maps, pooling operations are applied using different types of operations (Max, Sum, Average). Finally, the final convolutional layer output passes through a flatten operation to transform it into one vector, to be used later as input to the fully connected layer. The CNN is trained in a supervised manner, and the filters (kernels) are learned automatically during the training process of the CNN. The hyperparameters related to the kernels, such as their size, number, padding, and stride, need to be designed before the CNN training process. 

*Fully connected layer*: This is a fully connected feed-forward neural network that consists of a vector of neurons that are connected to all the neurons in the next layer through learnable weights. It receives the output of the flattening step, which is the last pooling step of the last convolutional layer.

*Output layer*: This includes an activation function, which is selected according to the type of classification task (e.g., binary classification: Sigmoid function, multiple class classification: Softmax function, continuous value regression: Linear function)

II. The Proposed CNN Architecture

In this work, the CNN architecture was chosen empirically by running multiple experiments with different architectures considering various numbers of layers and filters with different sizes and strides. 

**Figure 1:**
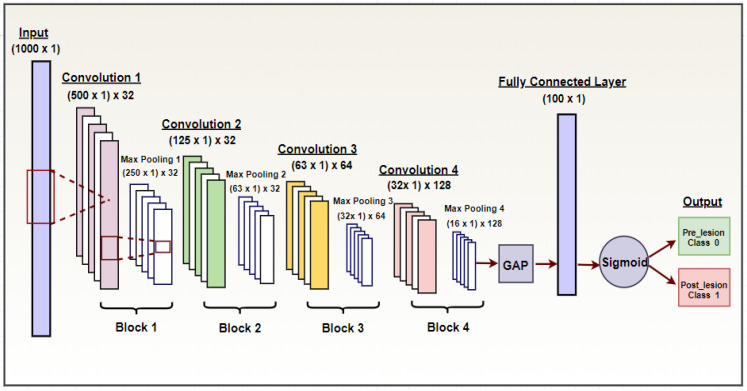
Convolutional neural network structure utilized to build the EMG-based TSCI classification system.

The final selected network consisted of five blocks, as illustrated in Figure 1 and Table 2. The CNN was structured as follows:

*Input:* An EMG segment (1000 × 1)

Block 1 was composed of:

- A convolutional layer with 32 filters having a size 5 × 1 and a stride of 2;
- An activation layer using rectified linear units (ReLUs) as activation functions;
- A sub-sampling layer (max-pooling).

Block 2 was composed of:

- A convolutional layer with 32 filters having a size of 5 × 1 and a stride of 2;
- An activation layer of ReLUs used as activation functions;
- A sub-sampling layer (max-pooling).

Block 3 was composed of:

- A convolutional layer with 64 filters having a size of 3 × 1 and a stride of 1;
- An activation layer of ReLUs used as activation functions;
- A sub-sampling layer (max-pooling), where a dropout of 0.1 was applied.

Block 4 was composed of:

- A convolutional layer with 128 filters having a size of 3 × 1 and a stride of 1;
- An activation layer of ReLUs used as activation functions;
- A sub-sampling layer (max-pooling), where a dropout of 0.1 was applied.

These four blocks were followed by:

A *global average pooling layer* (GAP), which computes the average value of each individual input feature map and yields a single feature map, obtained by concatenating the computed average values.

A *fully connected layer* (FC) of size 100 × 1, with ReLUs as activation functions.

An *output layer,* in which the sigmoid activation function was chosen to satisfy the binary classification requirement. This was used to classify the input data using the output from the FC layer into either the pre-lesion class (<0.5) or post-lesion class (>0.5).

## 3. Results and Discussion

The results of the classification comparison analysis are as follows:

### 3.1. CNN Hyperparameters

To design a neural network, certain variables need to be set before optimizing the network weights. These variables are known as network hyperparameters, and include variables such as the number of layers, the number of nodes, the learning rate, the batch size, and the number of epochs. We applied a manual search method to find the combination of hyperparameters that provided the best classification performance. Various combinations of the hyperparameters were evaluated, and the best combination was selected to perform the classification task. The hyperparameters were optimized using training and test sets, and the final results are reported using five-fold cross validation. The same procedure was applied to the EMG data for the right and left sides. Table 3 summarizes the selected hyperparameters.

The network was trained using the extracted EMG segments, along with their corresponding class labels (pre-lesion = Class 0, post-lesion = Class 1). The binary cross-entropy (BC) loss function was used, as shown in the following equation:

(1) $$\mathrm{BC}=-\frac{1}{n}\sum_{i=1}^{n}x_{i}\;.\;\log\left(y_{i}\right)+\left(1-x_{i}\right).\log\left(1-y_{i}\right),$$

where *y_i_* is the predicted probability, *x_i_* is the actual probability (0 or 1), and *n* is the total number of instances. All the experiments were conducted using a free cloud service based on Jupyter Notebooks, known as Google Colaboratory (Colab), which has been shown to be an effective tool for deep learning [34]. The CNN was implemented in Keras (a Python front-end for deep learning) [35], and the Python Tensorflow library [36] was used for the computational implementations.

### 3.2. CNN Over-Fitting

**Figure 2:**
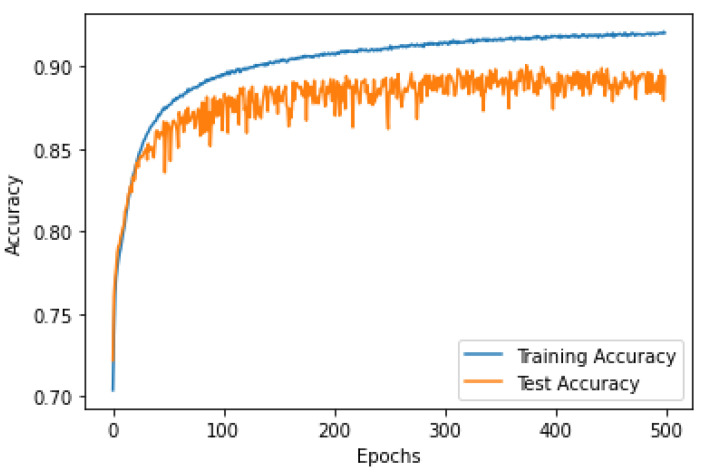
The accuracy curve for the EMG data classification of the left side using the proposed CNN architecture.

**Figure 3:**
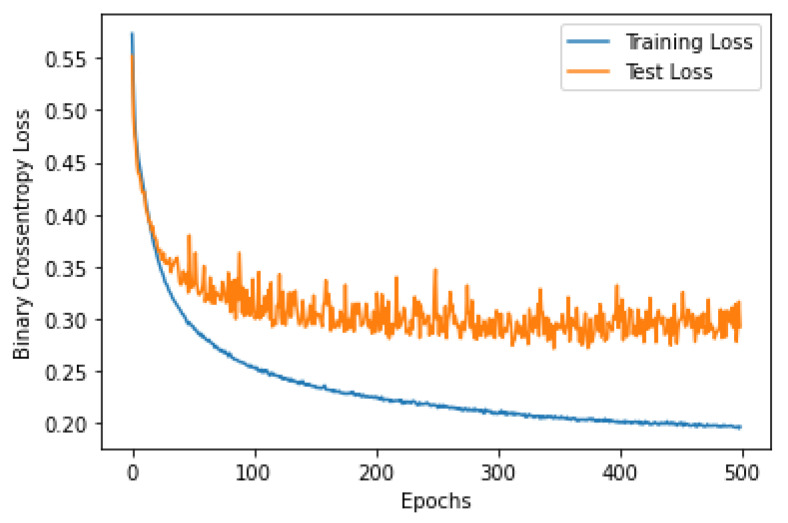
The loss curve for the EMG data classification of the left side using the proposed CNN architecture.

**Figure 4:**
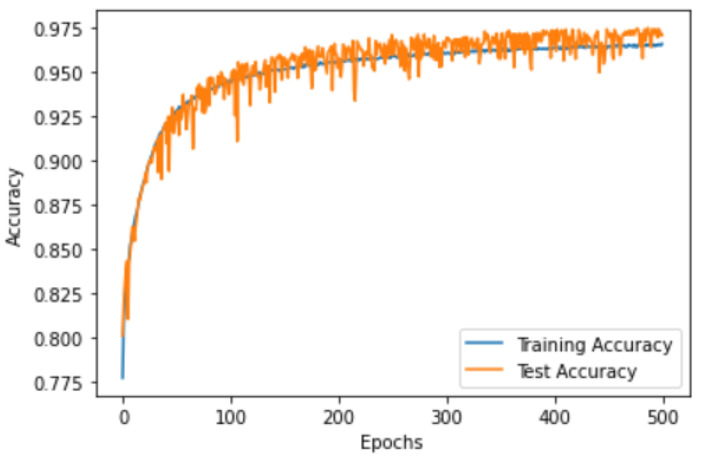
The accuracy curve for the EMG data classification of the right side using the proposed CNN architecture.

**Figure 5:**
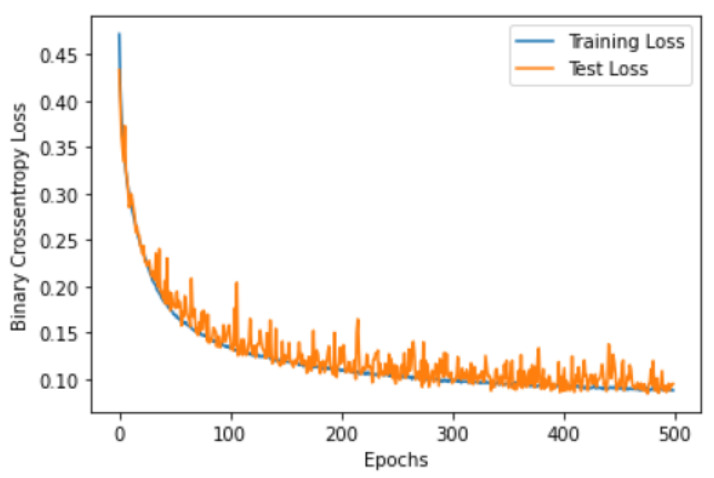
The loss curve for the EMG data classification of the right side using the proposed CNN architecture.

Over-fitting is a common problem in machine learning, which occurs when the model is too complex and fits the training data very well (memorizing the data). To test the performance of the proposed CNN classifier, the loss and the accuracy curves were visualized for both sides, when trained using 80% of the EMG segments and tested using 20% of the segments. Figure 2, Figure 3, Figure 4 and Figure 5 show the accuracy and the loss curves of the CNN for the left and right sides. In these figures, both the training and testing loss decreased continuously, and were close throughout the training process, which indicated that the proposed CNN did not face an over-fitting problem.

### 3.3. Performance Metrics of kNN and CNN Classification

The major goal of biological system statistical analysis is to obtain inference regarding the data generalization process by creating a mathematical model. Such a model will help in confirming previous biological knowledge about the studied system, as well as offering a suitable tool to test different hypotheses regarding the system behavior. Generally, statistical analysis requires data collected through controlled experiment design and a small–moderate sample size (compared to ML). Consequently, the statistical results and inferences would be more complex and less precise when dealing with complex and wide data [12]. On the other hand, artificial intelligence (AI) analysis (i.e., ML or DL) aims mainly to generate a prediction about unseen (unobserved) data, or to make a prediction about future behavior without requiring an understanding of the underlying mechanism (e.g., identifying the best course of treatment) [12]. AI analysis can be effective even with an unstructured data set collected in uncontrolled experiment settings, or data characterized by a complicated non-linear nature. AI techniques, including ML and DL, make minimal assumptions regarding the data generating system [12]. Hence, the classification techniques that were implemented in this study (CNN and *k*NN) might be a more suitable choice for developing a reliable assessment tool (automatic identification) [13] of TSCI using EMG signals.

**Figure 6:**
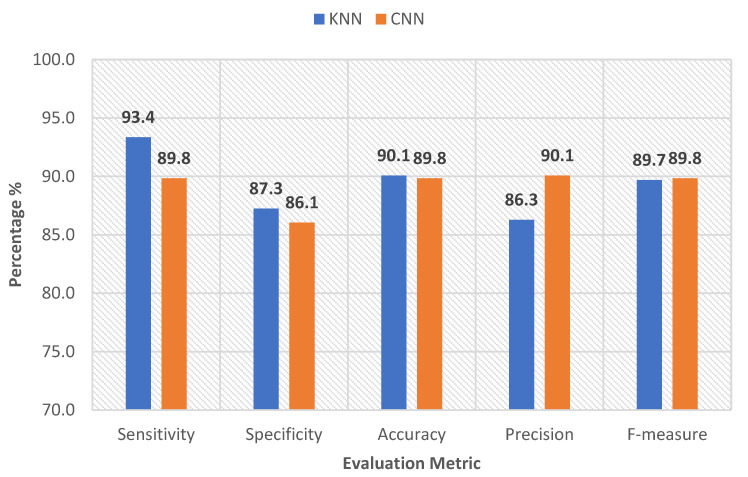
Evaluation metrics of the CNN and *k*NN classifiers (left side).

**Figure 7:**
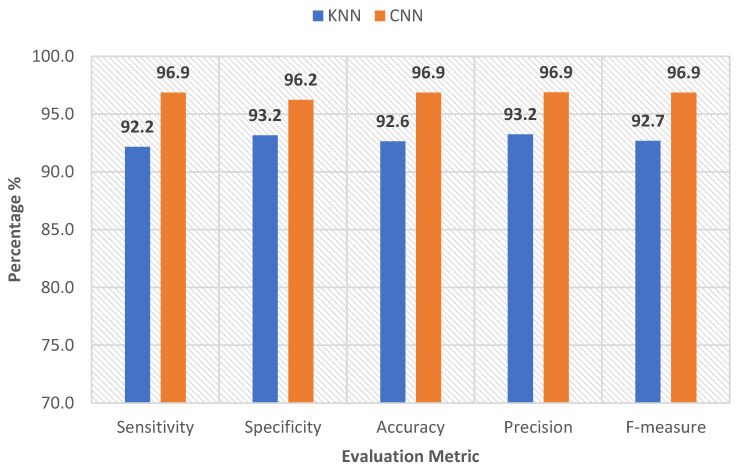
Evaluation metrics of the CNN and *k*NN classifiers (right side).

Figure 6 and Figure 7 show the five statistical metrics calculated for the *k*NN and CNN classifications of the EMG data for the muscles of the left and right sides, separately.

According to these figures, the *k*NN classified the EMG data with an F-measure of 89.7% for the left side and 92.7% for the right side. The simple proposed architecture of the CNN also performed reliably, as it achieved a higher F-measure of 89.8% and 96.9% for the left and right sides, respectively. The CNN model achieved a competitive result for all of the evaluation metrics for both sides. This can be explained by the advantage that the CNN has, in terms of its ability to use the temporal correlation existing inside the EMG segments. These results might also indicate that CNN had learned the required information from the EMG signal, reflecting the effect of the created lesions on the muscle activity. These unique EMG features were learned through the successive convolutional layers.

Therefore, such a type of learning system might help in interpreting the complex neural signals between the motor control system and the skeletal muscles, recorded as EMG signals. It may also help to characterize the neuromuscular abnormalities (i.e., perturbations in the electrical activity of the muscle) that occur as a result of neuromuscular diseases, including TSCI. Electromyography is a reliable and cost-efficient muscle activity assessment tool; however, due to the complex process of generating this signal, analyzing and understanding such a complicated signal is a challenging task. Notably, in this project, the EMG signal presented higher complexity as it was collected while the subjects were performing their daily activities without any restrictions (freestyle movement). The TSCI classification system could be applied in future work using a CNN system with a more advanced structure.

## 4. Conclusions

This study presented a new application of deep learning (CNN) as a TSCI classification system, and compared the results obtained with the proposed model with those of a classical machine learning classifier (*k*NN). The CNN classification system was employed using EMG segments as inputs, while the *k*NN was applied using four EMG hand-crafted features (i.e., area, RMS, turn number, and zero-crossing number). The performance of the two classifiers was measured and compared according to five performance metrics (accuracy, sensitivity, specificity, precision, and F-measure). From the obtained results, we found that the proposed CNN technique with automatically learned features achieved a competitive degree of classification accuracy, when compared to the classical *k*NN classifier. As the CNN technique is still a new application, more investigations need to be performed in order to obtain a clear understanding of the ability and reliability of such a deep learning technique. The work contributes to the literature and will help researchers learn about the effect of implementing the CNN for such a TSCI data set.

## Figures and Tables

**Table 1:** Previous works related to EMG signals and deep learning techniques.

#	Month/ Year	Study	DL Model	EMG Feature	Application	Conclusion
1	June/2019	[14]	Improved dual parallel channels convolutional neural network (IDPC-CNN).	Spectral features of sEMG	Fall detection using sEMG	IDPC-CNN was significantly better than other applied methods
2	May/2019	[15]	CNN	Spectrogram images	Pattern recognition	CNN classification accuracy of 88.04%
3	August/2018	[16]	Regression CNN	Raw EMG signal	Simultaneous EMG control	CNN-based system outperformed SVM-based system
4	August/2018	[16]	Compact CNN	Raw EMG signal	Gesture classification	CNN outperformed SVM
5	January/2019	[17]	CNN + transfer learning	Raw EMG, spectrograms, and continuous wavelet transform (CWT)	Gesture classification	Transfer learning enhanced the performance of the CWT-based CNN
6	October/2017	[18]	CNN	sEMG	Gesture recognition	CNN achieved an average accuracy of 97.81%
7	July/2017	[19]	CNN	sEMG	Pattern recognition	CNN showed a better performance compared to SVM
8	February/2017	[20]	CNN	sEMG images	Hand gesture recognition	CNN was better than LDA, SVM, *k*NN, and RF (state-of-the-art methods)
9	November/2016	[21]	CNN	sEMG images	Gesture recognition	CNN was better than LDA, SVM, *k*NN, MLP and RF
10	October/2016	[22]	CNN	sEMG spectrograms	Robotic arm guidance	CNN achieved state-of-the-art results
11	September/2016	[23]	CNN	Raw sEMG	Pattern recognition	CNN produced accurate results with a simple architecture, compared to the classical methods

**Table 2:** Optimized CNN architecture.

No	Architecture Parameters	Output Shape	No. of Parameters
1	CONV1: 32 × (5,1), stride: (2)	500 × 32	192
2	Maxpool1: (2,1), stride: (2)	250 × 32	0
3	CONV2: 32 × (5,1), stride: (2)	125 × 32	5152
4	Maxpool2: (2,1), stride: (2)	63 × 32	0
5	CONV3: 64 × (3,1), stride: (1)	63 × 64	6208
6	Maxpool3: (2,1), stride: (2)	32 × 64	0
7	Dropout3: (0.1)	32 × 64	0
8	CONV4: 128 × (3,1), stride: (1)	32 × 128	24,704
9	Maxpool4: (2,1), stride: (2)	16 × 128	0
10	Dropout4: (0.1)	16 × 128	0
11	Global Average Pooling	128	0
12	FC: 100 (ReLU)	100	12,900
13	Output: 1 (Sigmoid)	1	101
Total No. of Parameters			49,257

**Table 3:** The utilized hyperparameters.

Optimizer	Learning Rate	Batch Size	Epoch	Loss Function
Adam	0.001	128	500	BC

## Data Availability

The data is continuing to be analyzed by the research team and is currently not publicly available.
